# Distribution of common pathogens in patients with pyogenic liver abscess in China: a meta-analysis

**DOI:** 10.1007/s10096-016-2712-y

**Published:** 2016-07-11

**Authors:** M. Luo, X.-X. Yang, B. Tan, X.-P. Zhou, H.-M. Xia, J. Xue, X. Xu, Y. Qing, C.-R. Li, J.-F. Qiu, Y.-L. Li

**Affiliations:** 1 School of Public Health and Management, Chongqing Medical University, Chongqing, 400016 China; 2 Yubei District Center for Disease Control and Prevention, Chongqing, 401120 China; 3 School of Medicine, Johns Hopkins University, Baltimore, MD USA

## Abstract

Pyogenic liver abscess (PLA) is a potentially life-threatening disease in many parts of the world, especially in Asia. The aim of this study was to quantify the proportion of common pathogens in patients with PLA in China, using a meta-analysis method based on systematic review of published studies. Several electronic databases were searched to identify the studies reporting the pathogens of PLA. We performed a meta-analysis to calculate the pooled proportion of pathogens and subgroup analysis among the included studies using R 3.1.1 software. In total, 183 studies were included in our final analysis, *Klebsiella* spp (54 %), *Escherichia* spp (29 %), *Enterobacter* spp (9 %), *Proteus* spp (6 %) and *Pseudomonas* spp (5 %) comprised the major gram-negative bacteria. Gram-positive bacteria mainly included *Staphylococcus* spp (13 %), *Streptococcus* spp (8 %) and *Enterococcus* spp (7 %). The distribution of pathogens in PLA patients were different in different economic regions in China. The proportion of *Klebsiella* spp had an upward tendency in recent years compared to other pathogens. In addition, the proportion of common pathogens in PLA patients with diabetes mellitus (DM) were carried out indicating that the dominant pathogens were *Klebsiella* spp (66 %), *Escherichia* spp (21 %) and *Enterobacter* spp (11 %). This meta-analysis showed that the main pathogens of PLA were *Klebsiella* spp, *Escherichia* spp, *Staphylococcus* spp, and *Enterobacter* spp in China. To ensure a precise estimate of the epidemiology of the pathogens, further large-scale or even a population-based study is needed.

## Introduction

Pyogenic liver abscess (PLA) is a potentially life-threatening disease, which has been reported in many countries [1–7]. The morbidity of PLA has been increasing steadily in the past few decades, and although the mortality decreased slightly, PLA remains a disease with significant mortality [1, 5, 8–11]. Among others, surgical intervention and appropriate antibiotics combined with the US-guided percutaneous drainage of liver abscess constitute major treatments and increase the chances of survival [12, 13]. The diagnosis of PLA is based on clinical features, evidence from imaging studies and microbiology. Unfortunately, the diagnosis of PLA is technically challenging, due to the fact that the symptom presentation is non-specific [14]. The disease was more prone to run a lethal course if without appropriate treatment. Accurate identification of pathogens was important for early appropriate patient management. There is a chance of false negative detection when the patient received prior antibiotic treatments. When the diagnosis of PLA was suspected, broad-spectrum antibiotics will be started immediately to control ongoing bacteremia and its associated catastrophic complications including metastatic endophthalmitis and central nervous system infections [12, 15, 16]. Knowing the etiology of PLA, when possible, plays an important role in the successful therapy of PLA patients. The pathogens causing PLA are geographically diverse [1, 2, 11, 17]. In China, the major pathogen causing PLA is *Klebsiella pneumoniae* (*K. pneumoniae*) [5, 8]. To date, besides *K. pneumoniae*, other bacterial etiologies of PLA were lacking large epidemiological data. Diabetes mellitus (DM) is at increased risk for common infections [18, 19] and is a well-known risk factor for PLA [4], with reported co-existence rates of 48 % in Taiwan [20]. However, it is unclear whether there is a difference in the pathogens in PLA patients with DM. In order to summarize the distribution of pathogens of PLA in China, this would be used to guide clinicians in therapy decisions and the direction of the future research. We systematically searched the literature about the pathogens of PLA, described the main pathogens of PLA, the distribution of pathogens in different economic regions, the change trend of pathogens in recent years and the common pathogens in PLA patients with DM.

## Materials and methods

### Search strategy

We searched through the electronic databases PubMed, EMBASE, Highwire, Web of Science, China National Knowledge Infrastructure (CNKI), China Biological Medical Database (CBM), VIP Database for Chinese Technical Periodical (VIP) and Wanfang database using the following search terms: “liver abscess, hepatic disease, hepatic abscess, welling-abscess of the liver” and “etiology, pathogen, pathogens, pathogenic microorganisms, bacteria”. The references were also manually searched to identify additional relevant publications. All studies related to the pathogens of PLA were published from January 1, 2004 to March 1, 2015, with a second search done on September 20, 2015. We restricted our search to publications in English and Chinese.

Studies were included if: (1) The study was about PLA and pathogens, (2) The diagnosis of PLA was based upon clinical features, evidence from imaging studies (ultrasound or computed tomography), as well as microbiology (blood or abscess culture results); Liver abscess with demonstrated positive amoebic or hydatid serology were excluded [14], (3) Patients with diabetes were diagnosed according to World Health Organization (WHO) criteria [21], (4) The study regions were in China. The following studies were excluded: (1) Identical studies retrieved through different databases, (2) Editorials, reviews or conference articles, (3) The articles had incomplete information to calculate the proportion of pathogens.

### Study selection and data extraction

Based on these explicit criteria, two researchers independently performed the initial screening of title and abstract, whereby disagreements were resolved by reaching a consensus. These original articles were then retrieved and the full text screened for final inclusion and data extraction.

Two researchers independently extracted data from all of the included studies with Excel 2007 (Microsoft Corp, Redmond, WA). For each accepted study, the following data were abstracted: first author, study region and time, year of publication, sample size, methods of specimens’ cultures, the number and type of positive strains. The different kinds of strains were combined on the basis of Bergey’s Manual of Systematic Bacteriology.

### Statistical analysis

The pooled proportion of pathogens and their 95 % confidence intervals (CIs) were used in our study. All statistical analysis was conducted using the meta package of R 3.1.1 (Bell Laboratories); metaprop, metabias and metainf were chosen for analysis. The logit transformation was chosen to calculate the pooled proportion of pathogens among the included studies. Subgroup analysis focused on different economic regions and years. Heterogeneity was assessed by Q-test and *I*^*2*^ statistics [22]. If high and significant heterogeneity was detected across studies (*p* < 0.05, *I*^*2*^ > 50 %) a random effect model was used, otherwise, a fix effect model was used. Sensitivity analysis was performed to evaluate the stability of the meta-analysis result. Egger’s linear regression test [23] was used to statistically assess the publication bias (*p* > 0.05 was considered to have no publication bias).

## Results

### Study selection and characteristics

**Fig. 1:**
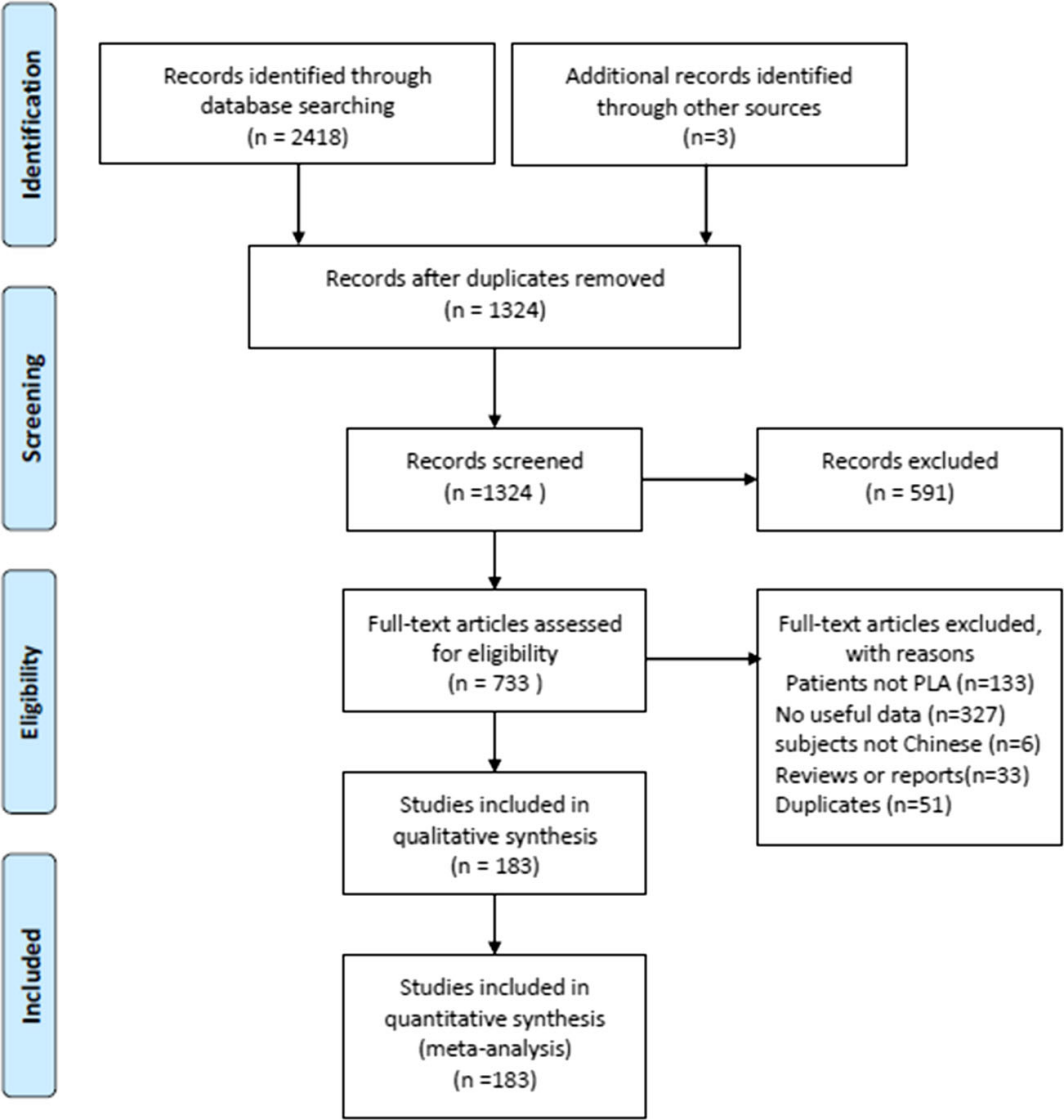
The PRISMA flowchart of the study identification and selection. *PLA* pyogenic liver abscess

A flowchart of the study identification and selection process was presented in Fig. 1. We identified 2,418 publications from eight electronic databases according to our search strategy and three publications from manually searched. A total of 1,097 publications were excluded due to duplicate publications. After screening of title and abstract, 591 publications were excluded. A total of 550 studies were excluded after full-text screening due to lack of adequate data, with subjects not Chinese, reviews or reports, duplicates, or not in accord with the diagnostic criteria of PLA. Finally, 183 studies were included in this meta-analysis, among them, 57 studies reported the pathogens of PLA patients with DM and were included in further analysis.

These 183 studies reported data on 6,913 strains of 6,347 subjects in the 31 provinces of China. Among them, six studies which included 1,215 patients with PLA were published in English, and the other studies were published in Chinese. There were no appropriate studies included in this meta-analysis in the three provinces: Macao, Qinghai and Tibet. The largest number of the included studies of province was Jiangsu (23 studies), followed by Zhejiang (20 studies), Shanghai (14 studies), Liaoning (13 studies), Beijing (11 studies) and so on. Sample size of those studies ranged from 2 to 301. The result of abscess culture was extracted in 71 % of the included studies, there were those with a small proportion (11 %) of the blood culture, and methods of specimen cultures were unknown accounted for 23 %. They were published from January 1, 2004 to September 20, 2015.

### The pooled proportion of pathogens

**Fig. 2:**
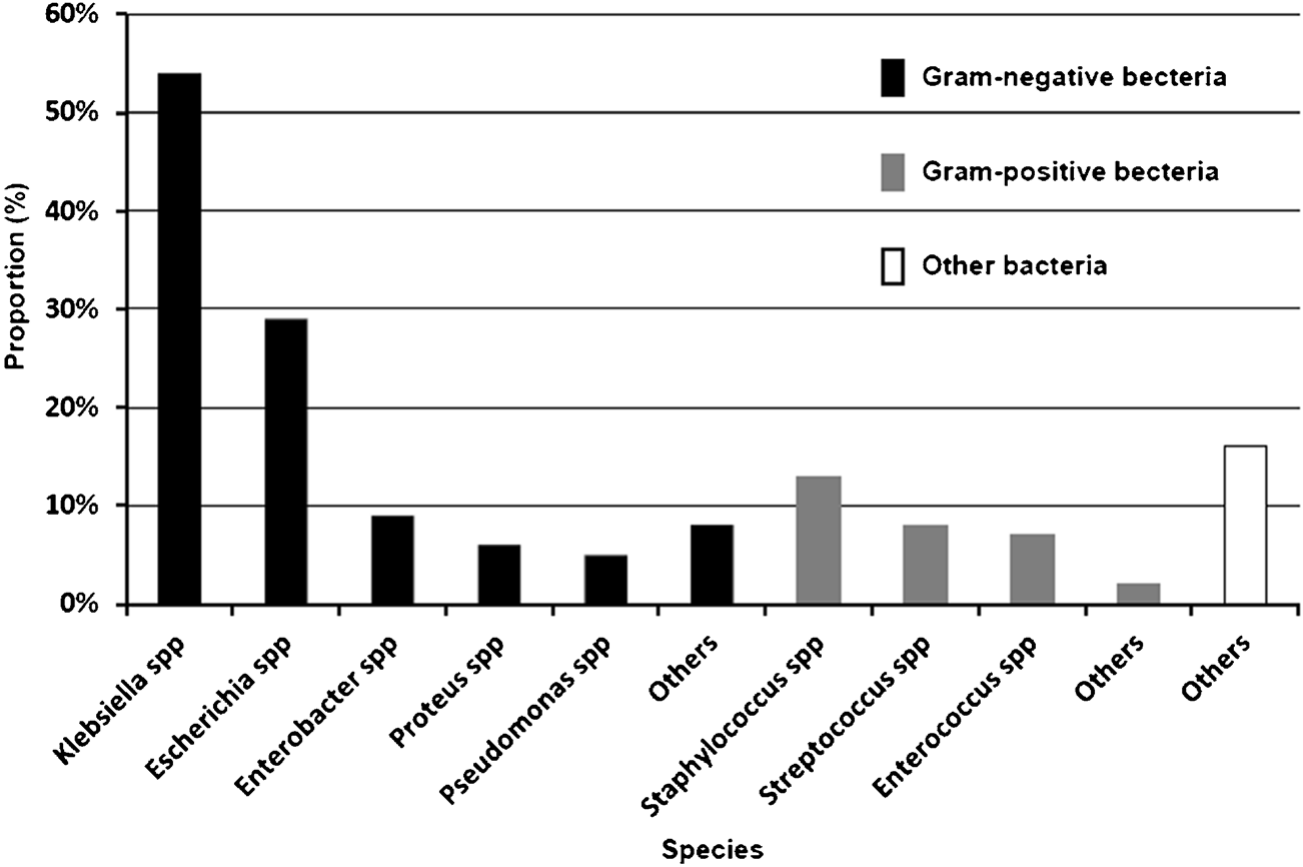
The histogram of common pathogens in patients with pyogenic liver abscess

For all included studies, when available, the proportion of pathogens was calculated and showed in Fig. 2 by meta-analysis methods with R 3.1.1 software. *Klebsiella* spp (54 %), *Escherichia* spp (29 %), *Enterobacter* spp (9 %), *Proteus* spp (6 %) and *Pseudomonas* spp (5 %) comprised the major gram-negative bacteria. The proportion of *Klebsiella* spp was much higher than that of other pathogens causing PLA. Gram-positive bacteria mainly included *Staphylococcus* spp (13 %), *Streptococcus* spp (8 %) and *Enterococcus* spp (7 %).

*K. pneumoniae* accounted for 93 % of *Klebsiella* spp and was the most common microorganism isolated from PLA patients. *Staphylococcus aureus* accounted for 88 % of *Streptococcus* spp and was the most common gram-positive bacteria, the specific bacteria was one of the other bacterial species not shown in this study. The proportion of other pathogens was less than 1 %, and we put them into other gram-negative bacteria or gram-positive bacteria. Some studies were only provided the number of pathogens and not the type, which we put into other bacteria.

### Distribution of pathogens in different economic regions

**Fig. 3:**
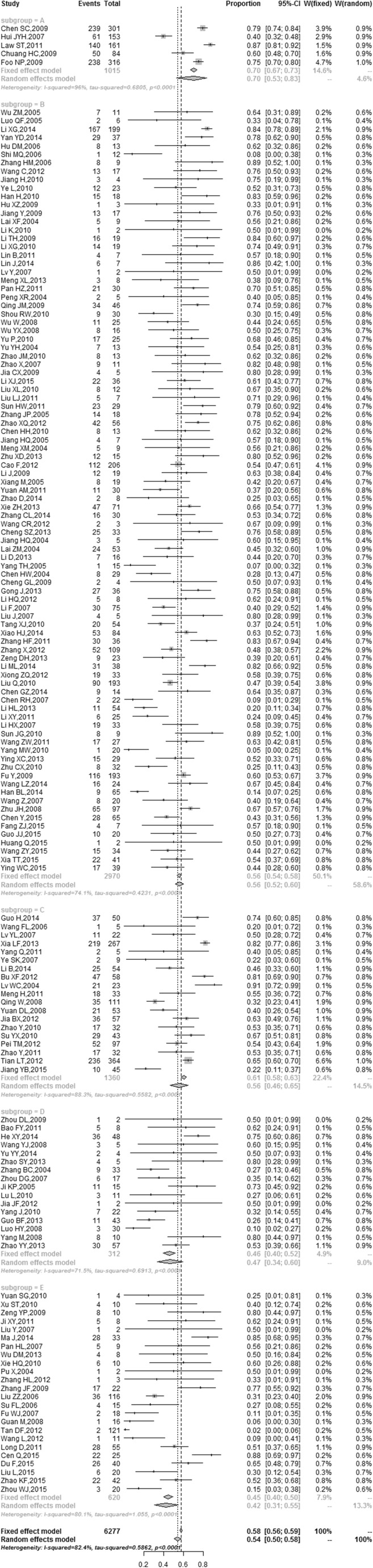
The Forest plot for the proportion of *Klebsiella* spp on different economic regions. The proportion of pathogens and its 95 % CIs were represented by a *diamond* and *horizontal line* in each study. Region A: Taiwan and Hong Kong, Region B: East economic region, Region C: Northeast economic region, Region D: Middle economic region, Region E: West economic region

We performed subgroup analysis in different economic regions with several common microorganisms. According to the conception of economic regional division during the 11th Five-Year Plan, the China mainland was divided into four economic regions, which included Northeast (Liaoning, Jilin and Heilongjiang), East (Beijing, Tianjin, Hebei, Shanghai, Jiangsu, Zhejiang, Fujian, Shandong, Guangdong and Hainan), Middle (Shanxi, Anhui, Jiangxi, Henan, Hubei and Hunan) and West regions (Inner Mongolia, Guangxi, Chongqing, Sichuan, Guizhou, Yunnan, Shaanxi, Gansu, Qinghai, Ningxia, Xinjiang and Tibet). We classified Taiwan and Hong Kong as another region. In total, China was divided into five regions. The GDP (gross domestic product) and GRP (gross regional product) per capita were used as indicators of economic status of each economic region. The annual data of GDP and GRP per capita of each province in China from 2004 to 2014 were retrieved by querying the National Bureau of Statistics of China website or from the yearbooks of the local Bureau of Statistics. According to the average of GRP per capita from high to low, the order of the economic regions would be the Region A: Taiwan and Hong Kong, Region B: East economic region, Region C: Northeast economic region, Region D: Middle economic region, Region E: West economic region. The economic status of five economic regions is shown in Table 1. We performed subgroup analysis with several common microorganisms in different economic regions. The Forest plot for the proportion of *Klebsiella* spp in different economic regions is shown in Fig. 3. The highest proportion of *Klebsiella* spp (70 %) was in the A region including Taiwan and Hong Kong, whereas the lowest was 42 % in the E region (West economic region).

**Table 1:** Economic status of five regions in China (the average from 2004 to 2014)

Economic region	Total GDP	GRP per capita
	(Hundred million RMB)	(RMB 10,000/person)
Northeast	11,517.86	3.11
East	21,252.84	4.61
Middle	13,235.57	2.23
West	6,365.48	2.19
Taiwan, Hong Kong	23,395.99	18.00

**Fig. 4:**
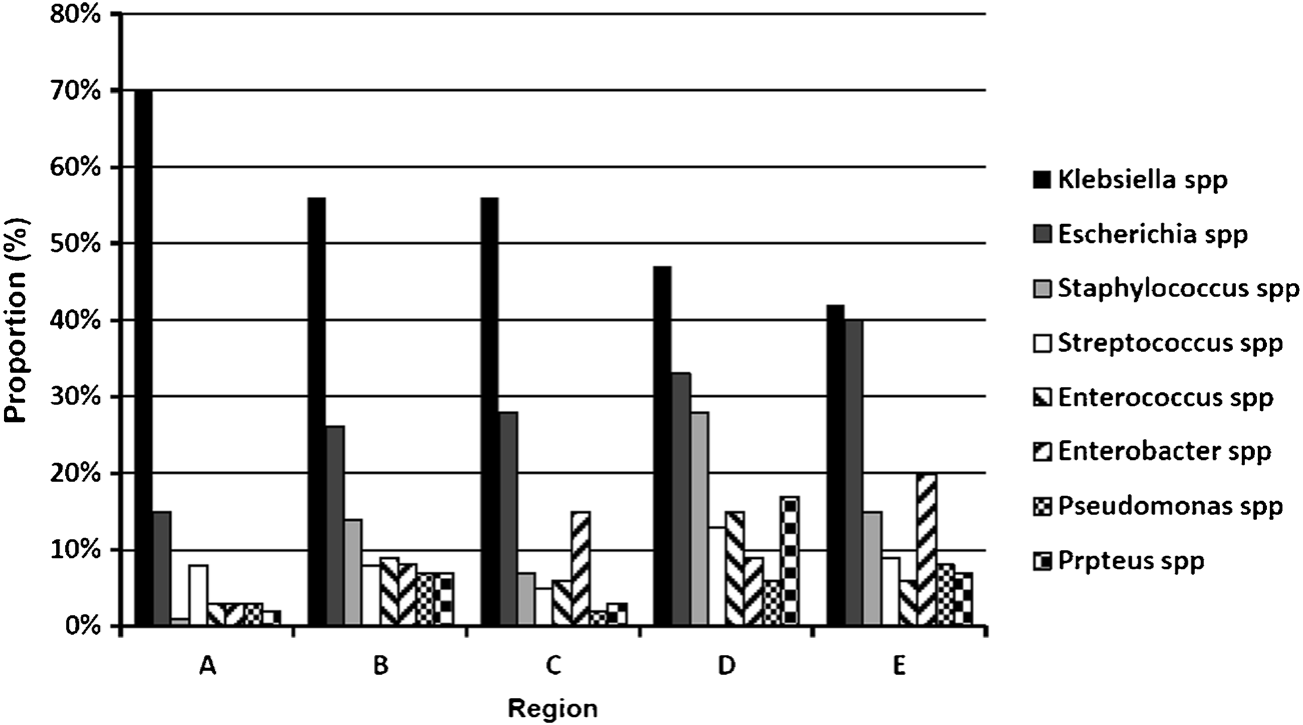
The histogram of pathogens of pyogenic liver abscess in different economic regions. Region A: Taiwan and Hong Kong, Region B: East economic region, Region C: Northeast economic region, Region D: Middle economic region, Region E: West economic region

Eight common pathogens of PLA in different economic regions were summarized in Fig. 4, which has been calculated separately by meta-analysis methods with R 3.1.1 software. The results indicated that *Klebsiella* spp, *Escherichia* spp and *Staphylococcus* spp were the main pathogens in the East and Middle economic regions; however, the dominant pathogens were *Klebsiella* spp, *Escherichia* spp and *Enterobacter* spp in the Northeast and West economic regions. In Taiwan and Hong Kong, these were *Klebsiella* spp, *Escherichia* spp and *Streptococcus* spp. The frequency of *Klebsiella* spp infection is shown in Fig. 4, which suggested that it was consistent with the level of regional economic development. However, lower economic regions have a higher frequency of *Escherichia* spp infection than the higher economic regions.

### The trend of pathogens in recent years

**Fig. 5:**
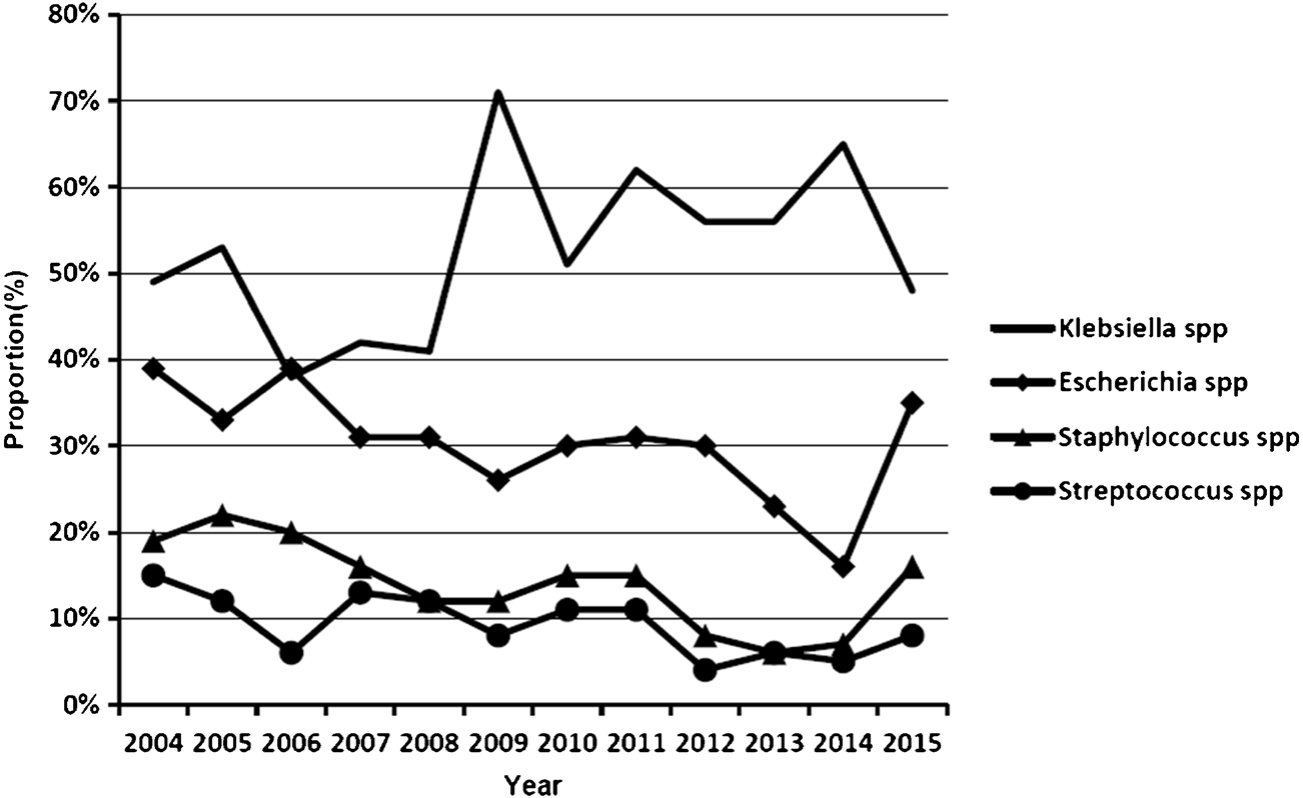
Change trend of pathogens of pyogenic liver abscess in recent years

We performed subgroup analysis in different years with four common microorganisms by meta-analysis methods with R 3.1.1 software, and the result is shown in Fig. 5. We did a search on September 20, 2015, so the data of pathogens in 2015 was incomplete. Between 2004 and 2015, the change trend of *Escherichia* spp, *Staphylococcus* spp and *Streptococcus* spp was stable, ranging from 16 % to 39 %, 6 % to 22 %, and 4 % to 15 %, respectively. Meanwhile, between 2004 and 2015, *Klebsiella* spp had an upward tendency, whereby the highest pooled proportion of *Klebsiella* spp was 71 % in 2009. The lowest was 38 % in 2006.

### The common pathogens in PLA patients with Diabetes mellitus

**Fig. 6:**
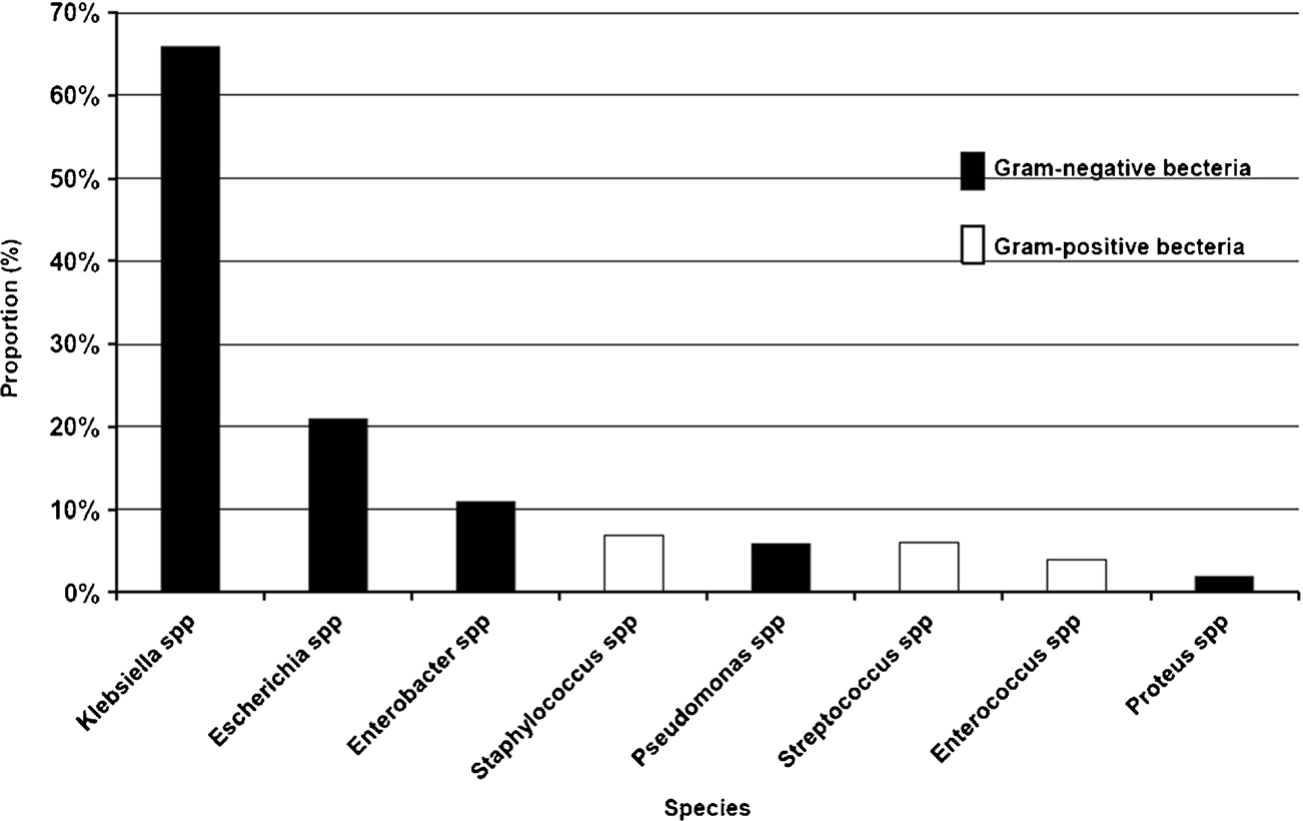
Histogram of the common pathogens in PLA patients with DM. *PLA* pyogenic liver abscess, *DM* diabetes mellitus. The pooled proportion of the pathogens are shown in Figure 6, where the results have been calculated separately by meta-analysis methods with R 3.1.1 software

We pooled the common pathogens of PLA patients with diabetes mellitus (DM). As shown in Fig. 6, the dominant pathogens were gram-negative bacteria including *Klebsiella* spp (66 %), *Escherichia* spp (21 %) and *Enterobacter* spp (11 %). There is a difference in the pathogens in PLA patients with DM. This was shown as the proportion of *Klebsiella* spp and *Enterobacter* spp were higher and that of *Escherichia* spp and *Staphylococcus* spp were lower. *Enterobacter* spp was more than *Staphylococcus* spp, which became the third most common pathogen in PLA patients with DM after those of *Klebsiella* spp and *Escherichia* spp.

### Sensitivity analysis and publication bias

**Fig. 7:**
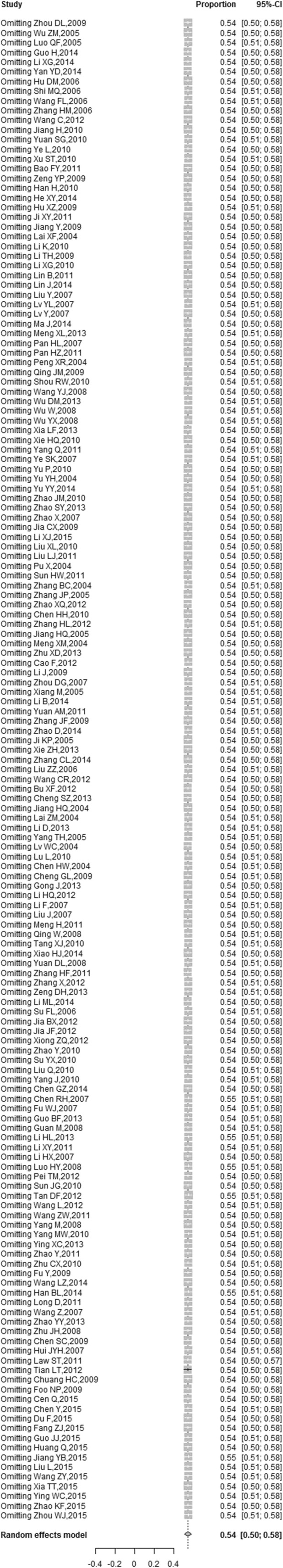
The Forest plot of sensitivity analysis of the proportion of *Klebsiella* spp. The pooled proportion of pathogens and its 95 % CIs were represented with a *diamond* and *horizontal line*

The sensitivity analysis results for eight different strains (*Klebsiella* spp, *Escherichia* spp, *Staphylococcus* spp, *Enterobacter* spp, *Streptococcus* spp, *Enterococcus* spp, *Proteus* spp, *Pseudomonas* spp) showed that the proportion of pathogens were not significantly different after omitting the individual study. One of the sensitivity analysis of *Klebsiella* spp is shown in Fig. 7.

Egger’s linear regression test was used to statistically assess the publication bias (*p* > 0.05 was considered to have no publication bias). The publication bias for eight different strains was performed. Three of them were found to have publication bias, namely, *Klebsiella* spp, *Escherichia* spp and *Staphylococcus* spp.

## Discussion

Pyogenic liver abscess (PLA) is a suppurative infection of the hepatic parenchyma, which can develop severe complications including metastatic endophthalmitis and central nervous system infections [15, 16], and patients with PLA have higher rates of primary liver cancer and gastrointestinal cancer [24, 25]. In China, the morbidity of PLA was increasing steadily in the past few decades. A comprehensive study on the pathogens of PLA has not been reported. A cross-sectional survey does not always reach this target due to its flaw in methodology [26]. Meta-analysis is a statistical method whereby data from a number of studies can be pooled to produce reliable data. This study was the first to demonstrate a relatively comprehensive evaluation of the pathogens of PLA in China. A total of 183 studies were enrolled by meta-analysis method, with inclusion of data from 91 % of the provinces in China. Although they did not provide drug sensitivity results for antibiotics, knowing the etiological distribution of PLA is informative in facilitating clinicians to identify appropriate treatments for this suspicious PLA patient with the negative results of bacteria cultures.

The available data suggested that *Klebsiella* spp was the most common microorganism isolated from PLA patients; after *Klebsiella* spp, *Escherichia* spp was the second pathogen causing PLA. In addition, *Staphylococcus* spp, *Enterobacter* spp, *Streptococcus* spp and *Enterococcus* spp were also common pathogens of PLA. It has been reported that the mortality rate of non- *K. pneumoniae* liver abscess was higher than that of *K. pneumonia* [27, 28]. Community-acquired methicillin resistant *Staphylococcus aureus* liver abscesses had been reported in Hong Kong [29]. In contrast to *K. pneumoniae*, we should also pay attention to other pathogens of PLA in order to reduce the mortality.

We further quantitatively estimated the proportion of pathogens in subgroups defined by regions and years. This meta-analysis indicated that the most common pathogens were *Klebsiella* spp in different economic regions. The proportion of *Klebsiella* spp is 70 % in Taiwan and Hong Kong. *K. pneumoniae* liver abscess (KPLA) is common [5] and has a high economic burden in Taiwan [30]. Fung [31] reported that intestinal colonization of virulent type *K. pneumoniae* is highly associated with PLA. Chinese ethnicity itself might be a major factor predisposing to intestinal colonization by *K. pneumoniae* [32]. It is probably the reason why there are so many cases of KPLA in China. It has been reported that diabetes mellitus (DM) is a more significant risk factor for the *K. pneumoniae* liver abscess than for the non-*Klebsiella* liver abscess [33]; the prevalence of DM was consistent with the level of regional economic development [34]. This may be one of the reasons why higher economic regions have a greater frequency of *klebsiella* infection than the lower economic groups. Additionally, Chen [35] reported KPLA are more prevalent in the cryptogenic liver abscesses. Future research should focus on the pathogenesis of KPLA to reduce the morbidity and economic burden.

This meta-analysis showed that *Klebsiella* spp had an upward tendency in China between 2004 and 2015. *K. pneumoniae* accounted for 93 % of *Klebsiella* species. The widespread multiple drug-resistance and hypervirulent variant K. pneumoniae (hvKP) infections have increasingly caused serious global public health concern [36]; two changing trends in *K. pneumoniae* infections have been reported in China [37–39]. The management of infections due to *K. pneumoniae* has been complicated by the emergence of antimicrobial resistance, especially to the carbapenems, since these agents are often the last line of effective therapy available for the treatment of infections caused by multidrug-resistant *K. pneumoniae* [40]. The epidemiology of new and dangerous hvKP strains has had some recent advances, yet overall it remains poorly understood [41]. Enhancing our understanding of those multiple drug-resistance *K. pneumoniae* and hvKP is critical to make treatment decisions for KPLA patients.

Based on these data, the pooled data suggested that the proportion of *Klebsiella* spp and *Enterobacter* spp were higher and that of *Escherichia* spp and *Staphylococcus* spp were lower in PLA patients with DM. It has been reported that DM is a more significant risk factor for the *K. pneumoniae* liver abscess than for the non-*Klebsiella* liver abscess [33]. This may be the reason why the proportion of *Klebsiella* spp was higher in PLA patients with DM. One animal study suggested that the hypermucoviscosity-negative *K. pneumoniae* may serve as an ideal model for identifying virulence factors under diabetic conditions [42], which is convenient in further study of the pathogenesis of diabetes patients who are more susceptible to getting KPLA. DM is a significant risk factor for developing metastatic infections from PLA [43], such metastatic endophthalmitis and central nervous system infections could lead to a devastating outcome without a prompt and appropriate management. Although DM shows a higher association with PLA, it does not seem to increase the mortality of PLA [20]. Some previous studies have revealed that gas-forming abscess, multi-drug resistant isolates, metastatic infection, and acute respiratory failure were associated with mortality of PLA [44, 45]. We need to identify other significant risk factors that increase the mortality of PLA; further studies should be followed to elucidate this issue.

The validity of original studies was influenced by many factors, which might affect meta-analysis [46]. Therefore, in these situations, combining data in a meta-analysis has to be performed with great care. However, there are still some limitations in our study. First, our study was limited by the incomplete collection of the pathogens data. Usually, a study has more than one specimens culture, the result including a more comprehensive number and type of pathogens than was extracted in our study, which causes inevitable loss of part of data. Furthermore, the pathogens of PLA include aerobic bacteria and anaerobic bacteria, the data of anaerobic bacteria was insufficient in the included studies, so we only reported the epidemiology of aerobic bacteria of PLA. Second, heterogeneity between studies still exists although we had a strict criterion to include the original literature. The sample size differs among the included studies, which may result in high heterogeneity. Third, there was publication bias in part of the reported outcomes. These cases reported from hospital were more than 90 % in this meta-analysis, and clinicians might be affected by subjective factors in reporting these cases, which might contribute to publication bias. Moreover, with no gold standard of diagnosis, we used a diagnostic criteria base on the literature of Pang [14], in which the definition of PLA was comprehensive according to clinical features, evidence from imaging studies and microbiology. Therefore, some studies were excluded because diagnostic criteria were unclear, which might contribute to publication bias. Nevertheless, the usual methods for assessing publication bias using statistical tests are only applicable to randomized controlled trials or trials with matched controls and not to observational studies [47, 48]. Therefore, the results that apply the formal assessments of publication bias to this analysis might be inappropriate.

In conclusion, the main pathogens of PLA were *Klebsiella* spp, *Escherichia* spp, *Staphylococcus* spp, *Enterobacter* spp, *Streptococcus* spp and *Enterococcus* spp in China. The proportion of *Klebsiella* spp is much higher than other pathogens, especially in PLA patients with diabetes mellitus. It is informative during the study of etiology of PLA and making timely treatment decisions for suspicious PLA patients with the negative results of bacteria cultures. At the same time, the future research should concentrate on the pathogenic mechanism of the main pathogens, the differential diagnosis of clinical pathological and physiological features, prognostic factors and so on, thus improving understanding of the burden of PLA. To ensure a precise estimate of the epidemiology of the pathogens, further large-scale or even a population-based study is needed.
